# A library of mammalian effector modules for synthetic morphology

**DOI:** 10.1186/1754-1611-8-26

**Published:** 2014-11-19

**Authors:** Elise Cachat, Weijia Liu, Peter Hohenstein, Jamie A Davies

**Affiliations:** University of Edinburgh, Centre for Integrative Physiology, Hugh Robson Building, George Square, Edinburgh, EH8 9XD UK; The Roslin Institute, University of Edinburgh, Easter Bush Campus, Midlothian, EH25 9RG UK

**Keywords:** Synthetic morphology, Synthetic biology, Morphogenesis, Morphogenetic effectors, Development, Human embryonic kidney cells

## Abstract

**Background:**

In mammalian development, the formation of most tissues is achieved by a relatively small repertoire of basic morphogenetic events (e.g. cell adhesion, locomotion, apoptosis, etc.), permutated in various sequences to form different tissues. Together with cell differentiation, these mechanisms allow populations of cells to organize themselves into defined geometries and structures, as simple embryos develop into complex organisms. The control of tissue morphogenesis by populations of engineered cells is a potentially very powerful but neglected aspect of synthetic biology.

**Results:**

We have assembled a modular library of synthetic morphogenetic driver genes to control (separately) mammalian cell adhesion, locomotion, fusion, proliferation and elective cell death. Here we describe this library and demonstrate its use in the T-REx-293 human cell line to induce each of these desired morphological behaviours on command.

**Conclusions:**

Building on from the simple test systems described here, we want to extend engineered control of morphogenetic cell behaviour to more complex 3D structures that can inform embryologists and may, in the future, be used in surgery and regenerative medicine, making synthetic morphology a powerful tool for developmental biology and tissue engineering.

**Electronic supplementary material:**

The online version of this article (doi:10.1186/1754-1611-8-26) contains supplementary material, which is available to authorized users.

## Background

Most efforts in synthetic biology have so far concentrated on the engineering of metabolic pathways or the engineering of sensory and logic systems, generally (although not exclusively) in unicellular organisms [1–5]. Anatomical structure, a feature as essential to complex animals as metabolism and logic, has so far received very little attention. However, the recent report by Carvalho *et al.* [6], who generated morphogen diffusion gradients from engineered cysts, illustrates the power of synthetic tools towards spatial engineering in mammalian cells. In recent essays, we have proposed the construction of genetic modules for ‘synthetic morphology’: programming mammalian cells to display specific morphogenetic behaviours using synthetic biology principles, the eventual aim being the self-organization of these cells into designer ‘tissues’ [7, 8].

The aim of synthetic morphology is greatly aided by the observation that most normal mammalian morphogenesis seems to take place using about ten basic cellular behaviours, each tissue using them in different sequences and to different extents [7, 9, 10]. Analytical approaches to developmental biology have created models (or at least narratives) of normal development [10], but it is difficult to test these models properly [11]. The usual method of inactivating a key component and verifying that the event fails to take place does not really test more than the necessity for particular components (see Davies 2009 for further discussion [12]). Synthetic biology offers new ways of studying complex systems by the bottom-up approach, i.e. dissecting a process into basic events/modules and combining them back up from scratch, building this process *de novo*, or at least part(s) of it. This way, engineered cells should be able to execute simple sequences of predetermined morphogenetic events and test theories of morphogenesis away from its complex, natural setting. The approach will also allow researchers to explore what cells *can* do, rather than merely what they normally do.

Careful review of the literature indicates that many basic morphogenetic behaviours can be triggered by the expression of a single protein [7], making the idea of constructing synthetic morphology modules a realistic proposition. Here we report the construction of five inducible effector modules, to control cell adhesion, elective cell death, cell fusion, cell locomotion and proliferation. These are produced in a standardized format, easy to connect to existing logic and sensory modules, and all have been made available in a public repository. We also report the testing of these synthetic, inducible modules in mammalian cells.

## Results

### Library construction

**Figure 1:**
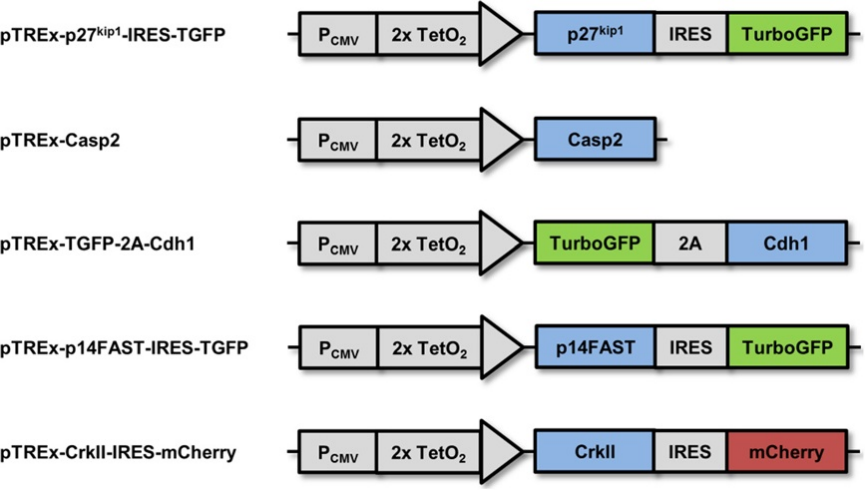
**The morphogenetic effectors used in this study.** *p27* ^*kip1*^ (control of proliferation), *Casp2* (elective cell death), *Cdh1* (cell-cell adhesion), *p14* (fusion) and *Crk-II* (locomotion) were inserted downstream of a tetracycline-responsive promoter and coupled with different fluorescent reporters in pTREx destination vectors.

We assembled a Gateway® cloning (Invitrogen) compatible pENTR library of morphogenetic effectors: these include *Cdh1* (cell-adhesion [13]), *Casp2* (elective cell death [14], *p14* (cell fusion [15]), *Crk-II* (cell locomotion [16]) and *p27*^*Kip1*^ (inhibition of otherwise constitutive proliferation [17]). The T-REx™-293 cell line (Invitrogen), a commercially available and easy-to-maintain cell line, was used to test these effector modules. It is derived from Human Embryonic Kidney (HEK) cells, to which stable expression of the tetracycline repressor protein has been added. T-REx-293 cells were used in combination with Gateway® pT-REx™-DEST vectors, so that driver genes could be conveniently inserted downstream of a tetracycline-inducible promoter from our library of pENTR library vectors. This way, effector modules could be switched on and off using tetracycline (Tet), without the need for construction and testing of the elaborate upstream logic modules that will control effector modules in advanced synthetic morphology projects [7, 8, 18]. After stable transfection, followed by clonal selection, independent clones were tested. Generally, effector genes were from a non-human origin in order to facilitate monitoring of their expression in T-REx-293 cells with species-specific antibodies or primers, the only exception being the gene encoding p27^Kip1^. Fluorescent reporters were also used in the system, by engineering pT-REx™-DEST vectors with IRES-TurboGFP or IRES-mCherry cassettes downstream of the effector, or by using 2A peptide fusions inside the effector cassette itself (Figure 1). Such reporters enable (i) the testing of clones for the simultaneous expression of the reporter and the gene of interest after stable selection and clonal isolation, (ii) real-time monitoring of inducible expression and (iii) visualization of different cell populations within a mixture of cells. Below, we demonstrate that each driver construct’s behaviour is effective in controlling the expected aspect of morphogenetic cell behaviour.

### Effector 1: control of proliferation

By definition, mammalian cell lines proliferate constitutively and indefinitely in culture [19]. In systems where proliferation of cells needs to be controlled, chemical inhibitors such as mitomycin C are used. A common example is the growth inhibition of feeder layer cells for the expansion of stem cells [20]. However, chemical inhibitors can metabolically alter cells [21] and are difficult to remove from culture systems [22]. In contrast, imposing proliferation control through genetic engineering would allow reversible, conditional and selective growth inhibition. This way, targeted populations of cells could selectively be controlled in systems or tissues composed of different cell types. Progression of cells through the cell cycle is regulated by complexes of cyclins with cyclin-dependent kinases (CDKs) [23]. Their action can be inhibited by cyclin-dependent kinase inhibitors (CDKIs), which are important in allowing cells to exit the cell cycle and to differentiate during foetal development [24]. p27^Kip1^ is a member of the CDKI family that binds G1-cyclin/CDK complexes and inhibits transition from the G1 into the S phase of the cell cycle [25]. Overexpression of p27^Kip1^ causes human fibroblasts to arrest in G1 phase [17]. To build an inducible, proliferation-inhibiting module, we placed *p27*^*Kip1*^ under tetracycline induction in the pTREx-DEST-IRES-TGFP modified vector (pTREx-p27^kip1^-IRES-TGFP, Figure 1).

**Figure 2:**
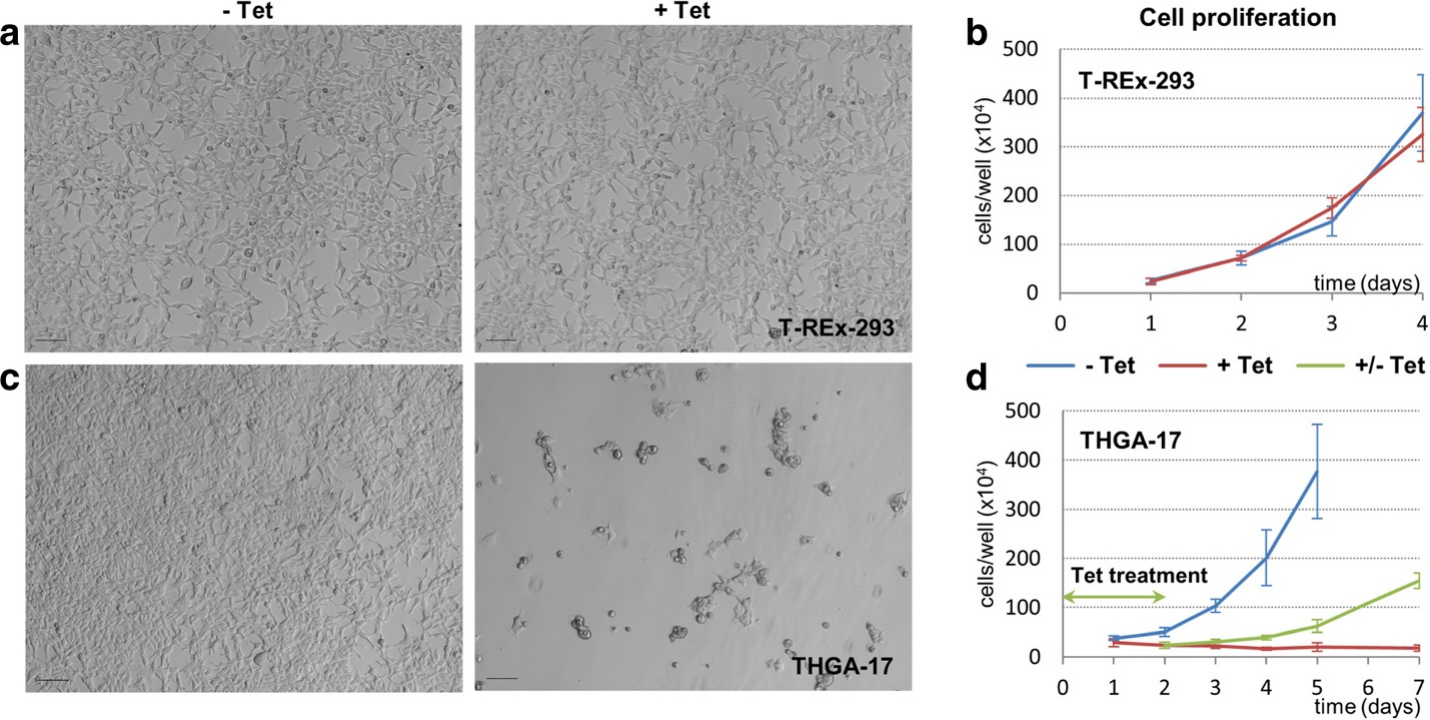
**Control of cell proliferation through p27** ^**kip1**^ **inducible expression. (a)** Control T-REx-293 cells were seeded in 6-well plates and cultured for 72 h with or without 0.05 μg/mL tetracycline. **(b)** Cell counts in triplicate wells showed that tetracycline itself had no effect on wild-type cell growth. **(c)** Cells from clone THGA-17 (a representative clone of T-REx-293 cells carrying the growth arrest module) showed clear growth rate differences after 72 h of culture with tetracycline. **(d)** After 48 h of growth inhibition with tetracycline, culture in tetracycline-free medium released THGA-17 cells from proliferation inhibition (green line). Scale bars: 100 μm. Standard deviation bars: n = 3.

Control T-REx-293 cells treated with 0.05 μg/mL tetracycline showed population growth rates similar to untreated wild-type cells (Figure 2a,b). On the other hand, clones stably transfected with *p27*^*Kip1*^ and induced with tetracycline for 72 h showed less proliferation than their uninduced (but still *p27*^*Kip1*^-carrying) counterparts (Figure 2c). Arrested cells appeared rounded and attached to the culture plate surface. It was important to test that the great reduction in proliferation that we observed was due to a reversible brake on cell cycling, and not as a result of cell damage or cell death. To verify this, we exploited the flexibility of the Tet induction system and observed the behaviour of cells following release from p27^Kip1^-mediated inhibition of proliferation. Cells were induced with tetracycline for 48 h, when the medium was replaced by the tetracycline-free version: released cells resumed proliferation after a delay of about one day (Figure 2d), although somewhat slower than their never-inhibited counterparts. Arrested cells could withstand tetracycline exposure for as long as 72 h before resuming growth. Longer exposures resulted in cell death.

### Effector 2: elective cell death

Elective cell death (as distinct from death due to infection or external injury) is an important mechanism of morphogenesis. It is used, amongst other things, to turn solid rods into hollow tubes [26], to eliminate temporary ‘scaffold’ structures [27] and to balance populations of different types of cells [28, 29]. The best-understood mechanism of elective cell death, apoptosis, is activated by caspases: these are cysteine proteases and are classified as initiator or effector caspases, according to where they lie in the apoptotic pathway. Caspase-2, unusually, shows characteristics of both initiator and effector caspases [30, 31]. It localizes to the nucleus where it is activated through dimerization [32]. It has been shown to induce apoptosis when overexpressed in NIH-3T3 mouse fibroblasts [14]. To engineer an elective cell death module, we placed mouse *Casp2* under tetracycline induction in the pT-REx-DEST30 vector (pTREx-Casp2, Figure 1).

**Figure 3:**
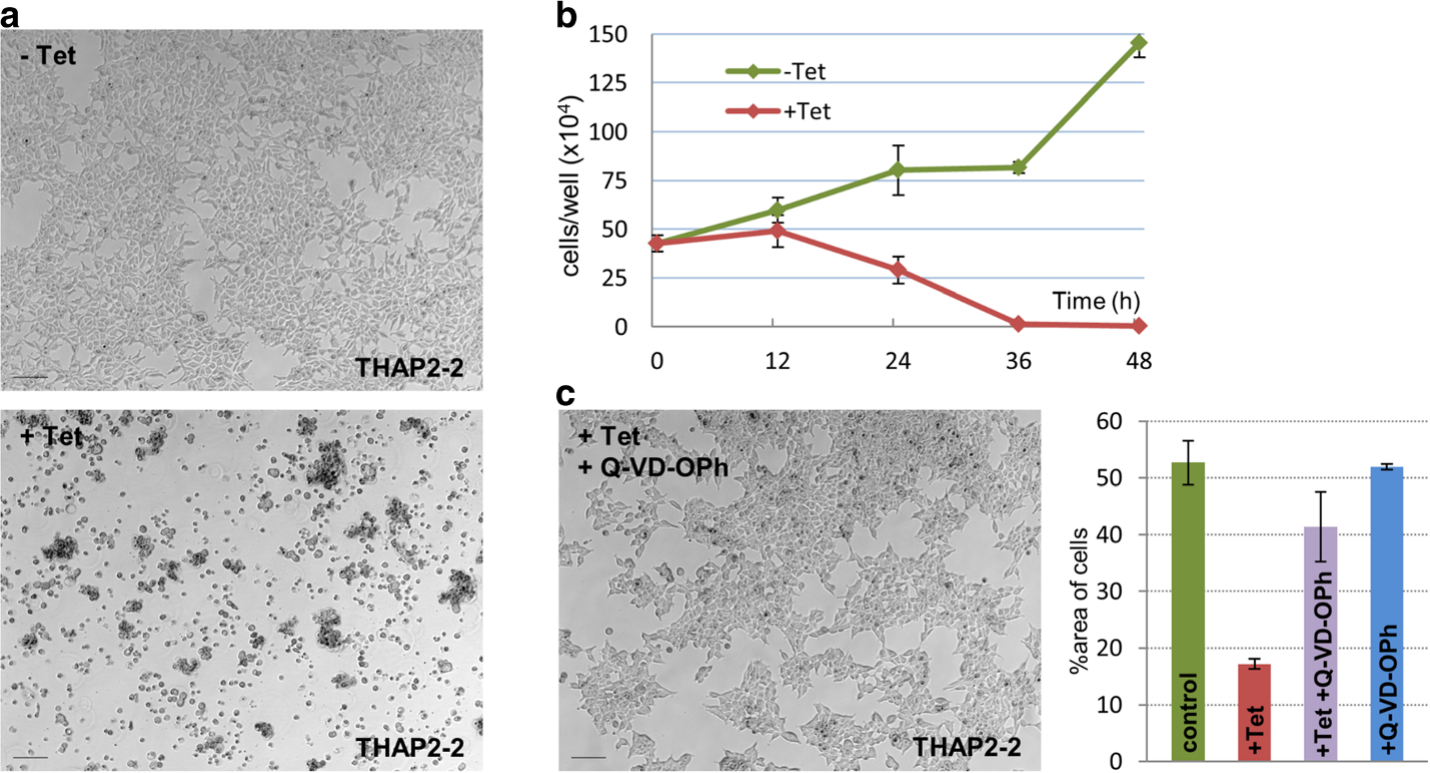
**Tetracycline-induced cell death in** ***Casp2*** **-engineered T-REx-293 cells. (a)** Uninduced cells from clone THAP2-2 (a representative clone of T-REx-293 cells carrying the elective cell death module) showed normal growth, but extensive cell death when induced with tetracycline (1 μg/mL) for 48 h. **(b)** Numbers of adherent, uninduced and induced THAP2-2 cells were monitored over 48 h after induction in 24-well plates. Standard deviation bars: n = 3. **(c)** When medium was supplemented with caspase inhibitor Q-VD-OPh (50 μM), cell death was largely blocked. Graph: percentages of area covered by adherent cells in various image fields: under normal growth conditions, after tetracycline induction, after tetracycline induction in the presence of Q-VD-OPh or in the presence of Q-V-D-OPh alone. Scale bars: 100 μm.

Uninduced T-REx-293 cells carrying the elective cell death module (Figure 3a, Additional file 1: Movie 1), as well as control wild-type T-REx-293 cells treated with tetracycline (see Figure 2a), showed little cell death and their number increased after 48 h in culture due to proliferation. After induction, the cells carrying pTREx-Casp2 showed extensive apoptotic death, detectable by microscopy (Figure 3a, Additional file 1: Movie 1) and by counting the declining number of cells still adhering to the wells (Figure 3b). To verify that the mechanism of cell death was that intended, we used Q-VD-OPh, a pan-caspase inhibitor. When added to the growth medium, it largely blocked cell death after tetracycline induction of the module (Figure 3c), confirming that the cell death observed previously (in absence of the inhibitor) was indeed caspase-driven.

### Effector 3: cell-cell adhesion

Arguably, the most important regulators of cell-cell adhesion in mammalian development are the various cadherins, a family of calcium-dependent adhesion molecules. The first to be discovered [33, 34], E-cadherin (CDH1), is a cell surface protein involved in intercellular adhesion of epithelial cells. This protein possesses a large extracellular domain that consists of repeated motifs responsible for homophilic adhesion between adjacent cells. In the cytoplasm, its intracellular domain binds to catenins and links E-cadherin to the actin cytoskeleton [35]. CDH1 promotes strong cell aggregation when expressed in mouse fibroblasts deficient in E-cadherin (L-929 cells) [13]. To build an adhesion module, we fused mouse *Cdh1* to TurboGFP through a 2A peptide link, and inserted the resulting cassette into pT-REx-DEST30 (pTREx-TGFP-2A-Cdh1, Figure 1).

**Figure 4:**
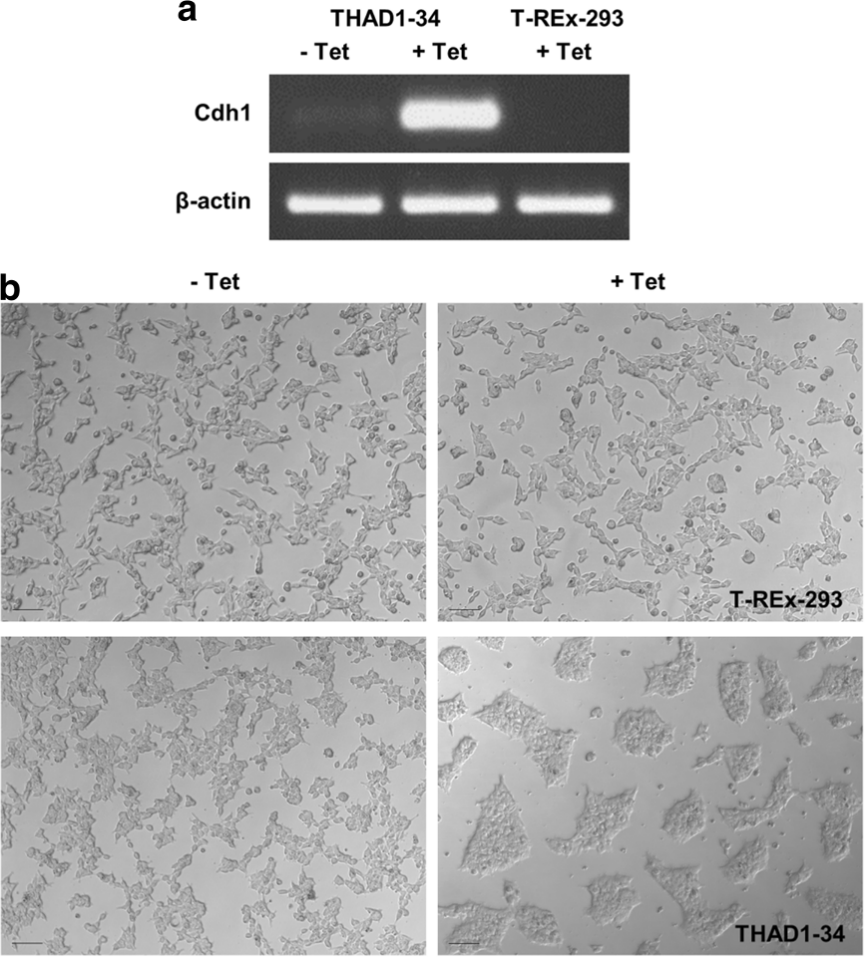
**Effect of the adhesion effector on engineered T-REx-293 cells. (a)** Levels of murine *Cdh1* mRNA expression in cells of clone THAD1-34 (a representative clone of T-REx-293 cells carrying the adhesion module), uninduced or induced with 1 μg/mL tetracycline for 48 h; and in wild-type T-REx-293 cells treated with tetracycline, as a control, for 48 h. **(b)** Morphology of wild-type T-REx-293 cells and THAD1-34 cells growing with or without tetracycline for 48 h: wild-type cells with or without tetracycline and uninduced THAD1-34 cells spread across the culture substrate with only loose mutual association, whereas induced THAD1-34 cells adhere to one another strongly to produce dense islands separated by clear space. Scale bars: 100 μm.

After clone selection, T-REx-293 cells stably transfected with pTREx-TGFP-2A-Cdh1 expressed mouse E-cadherin after 48 h of 1 μg/mL tetracycline induction, as shown by RT-PCR analysis (Figure 4a). The primers used to detect levels of *Cdh1* mRNA were specific for mouse *Cdh1*. When uninduced, cells show very little mouse E-cadherin mRNA transcription compared to induced cells, indicating a low level of expression leakage from the tetracycline transcriptional switch. Wild-type cells and T-REx-293 cells carrying the adhesion module but without induction showed only modest cell-cell adhesion: they grew in small, connected, ragged-edged clumps that formed a network across the culture plate (Figure 4b). Tetracycline itself had no effect on the morphology of wild-type T-REx-293 cultures. The same cells under tetracycline induction of *Cdh1* formed much more compact, dense, smooth-edged, separate islands (Figure 4b). These features are as expected, for increased adhesion would increase ‘surface tension’ at the edges of the islands, minimizing their circumference.

### Effector 4: fusion

Cell-cell fusion is used in embryonic events such as formation of skeletal muscle fibres [36]. For the purposes of synthetic biology, it is a potentially useful way to bring two different nuclei together in one cytoplasm, and thus to share cytoplasmic regulators of gene expression or metabolic effectors. The fusion-associated small transmembrane (FAST) proteins are reptilian reovirus membrane fusion proteins that are involved in virus dissemination in the host. They are expressed in the plasma membrane of infected cells where they mediate cell-cell fusion by converting pre-existing cellular adhesion sites into fusion sites [37]. Apposed membrane bilayers merge, hemifusion stalks appear, and pores are created and expand [15]. During viral infection, formation of the resulting extensive multinucleated syncytium eventually triggers an apoptotic response that contributes to the dissemination of infection in the host [38]. p14FAST, a reptilian orthoreovirus protein, is known to cause cell-cell fusion when expressed in QM5 quail fibrosarcoma cells [15], suggesting it will possibly function in mammalian cells too. To construct a fusion module, we placed *p14* under tetracycline induction in the pTREx-DEST-IRES-TGFP modified vector (pTREx-p14FAST-IRES-TGFP, Figure 1).

**Figure 5:**
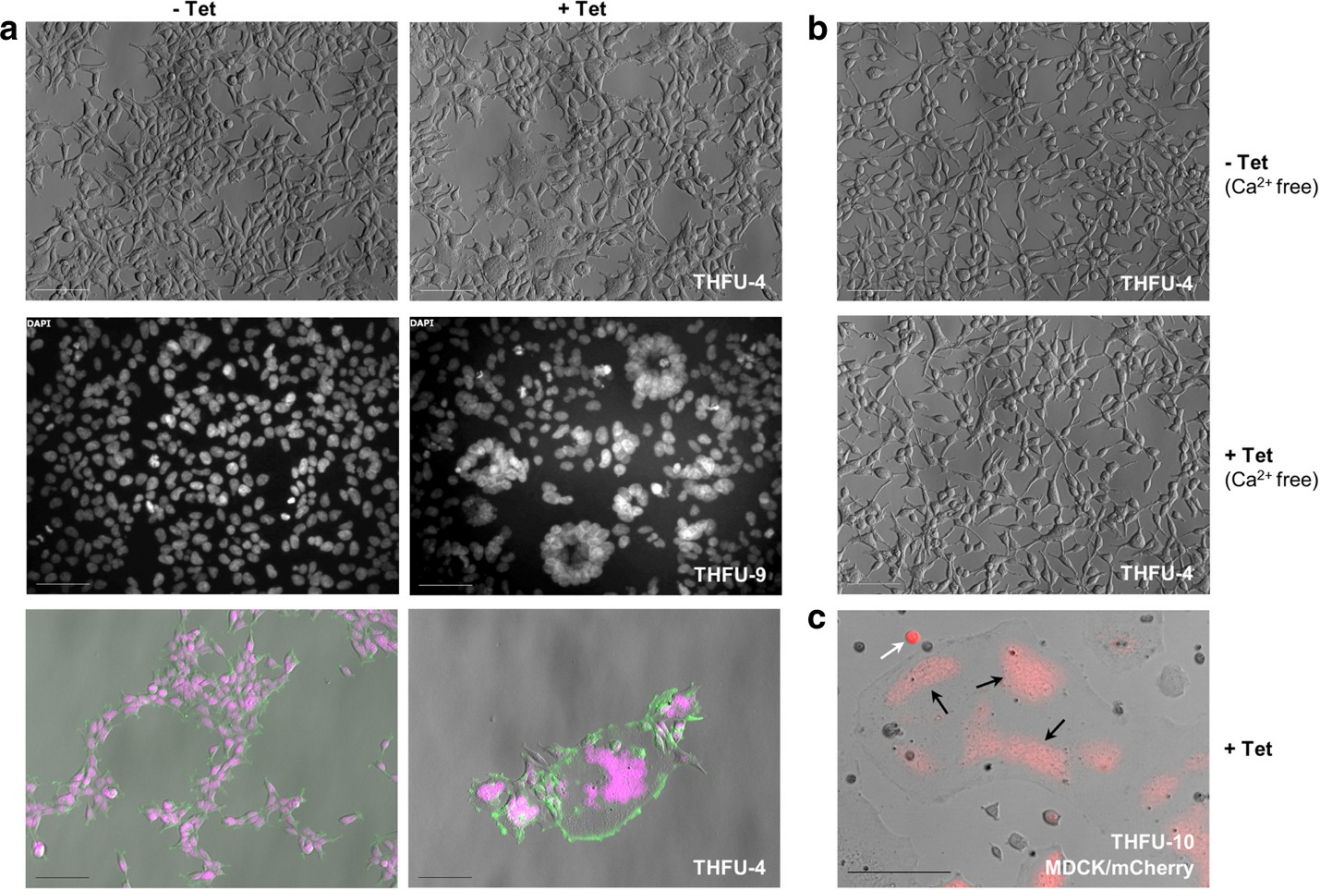
**Tetracycline-induced cell fusion in** ***p14*** **-engineered T-REx-293 cells. (a)** Bright-field microscopy, DAPI staining (white or magenta) and phalloidin-FITC (green) of THFU-4 cells (a representative clone of T-REx-293 cells carrying the fusion module), treated or not with tetracycline for 24 h. Untreated cells (left column) grow normally, without obvious signs of fusion, nuclei remaining separate and surrounded individually by cell membranes, shown by phalloidin stain of cortical actin filaments in the bottom row. Cells in which the fusion construct is induced show formation of large, multinucleate cells in bright field illumination (top row), formation of ‘rosettes’ of tightly apposed nuclei (middle row), with cortical actin (green) surrounding the whole groups of nuclei rather than each individual one (bottom row). **(b)** Cells grown in calcium-free medium showed fewer fusion events, as expected (*33*). **(c)** Evidence of fusion between tetracycline-induced cells from clone THFU-10 (another representative clone) and MDCK cells transiently expressing mCherry: unfused MDCK cells appear as small, deep red individual cells (white arrow). The red is also seen (black arrows) in large syncytia, implying that the THFU-10 cells can fuse with cells not themselves containing the fusion module. Scale bars: 100 μm.

Control T-REx-293 cells (see Figures 2a and 4b), and T-REx-293 cells carrying the fusion module but without induction (Figure 5a), showed no evidence of cell-cell fusion. Cells overexpressing p14FAST after tetracycline induction formed multinucleated syncytia as shown in Figure 5a: these could be detected as large, multinucleate cells in bright field that showed formation of tight ‘rosettes’ of nuclei visible with DAPI staining. In uninduced (unfused) cells, cortical actin surrounds each individual nucleus, indicating that the nucleus is in cytoplasm surrounded by its own plasma membrane: in the induced (fused cells), cortical actin instead surrounds complete rosettes of nuclei rather than individual ones, implying that the nuclei share one common cytoplasm. Syncytia formed through p14-induced fusion started dying around 20 h after tetracycline induction. Fusion events were rare when cells were grown in calcium-free medium (Figure 5b), confirming that the fusion process is calcium-dependent as expected [37]. To test whether the construct needed to be expressed in both neighbouring cells for fusion to take place, we mixed T-REx-293 cells carrying the fusion construct with MDCK canine kidney cells that had been transiently transfected with pTREx-mCherry as a marker (but not with the fusion construct). MDCK cells were chosen as they are well-characterized cells routinely used in our laboratory, and are from a different cell type and a different species than HEK cells (dog). They do not naturally form syncytia. In the mixed culture (Figure 5c), while some normal (unfused) MDCK cells could be seen as intensely red individual cells, some of the large syncytia contained red fluorescent patches suggesting that syncytia had formed in the mixed culture, and contained cytoplasms originating from both cell types. Potential users of this module should note that this result implies a risk that cells carrying the fusion module might fuse with cells of living humans, and potentially carry dangerous genetic material such as oncogenes and viruses into them (even basic cells lines carry mutations making them proliferative and immortal). Appropriate precautions should therefore be taken when handling the cultures.

### Effector 5: locomotion

Cell locomotion is a major mechanism in mammalian development: the long migrations of the neural crest and the primordial germ cells depend on it, as does wiring of the nervous system. Even in adult life, cells of the immune and inflammatory systems make constant use of locomotion. Cell migration is driven by the local polymerization of actin into protrusions called lamellipodia and filopodia, in response to a combination of internal cell state and external environmental cues [39]. These protrusions are stabilized by the formation of integrin adhesions to the extracellular matrix and extend in the direction of migration [40, 41]. Complex cytoskeletal reorganization also takes place at the rear of the cell, where existing adhesions are remodelled. CRK-II, an adaptor protein from cytoskeletal signalling pathways, has been shown to promote cell migration when expressed in human pancreatic carcinoma cells [16]. It promotes the formation of lamellipodia and the loss of cell-cell junctions (and consequent loss of cell-cell adhesion) in MDCK cells [42]. To engineer a module that promotes locomotion, we placed *Crk-II* under tetracycline induction in the pTREx-DEST-IRES-mCherry modified vector (pTREx-CrkII-IRES-mCherry, Figure 1).

**Figure 6:**
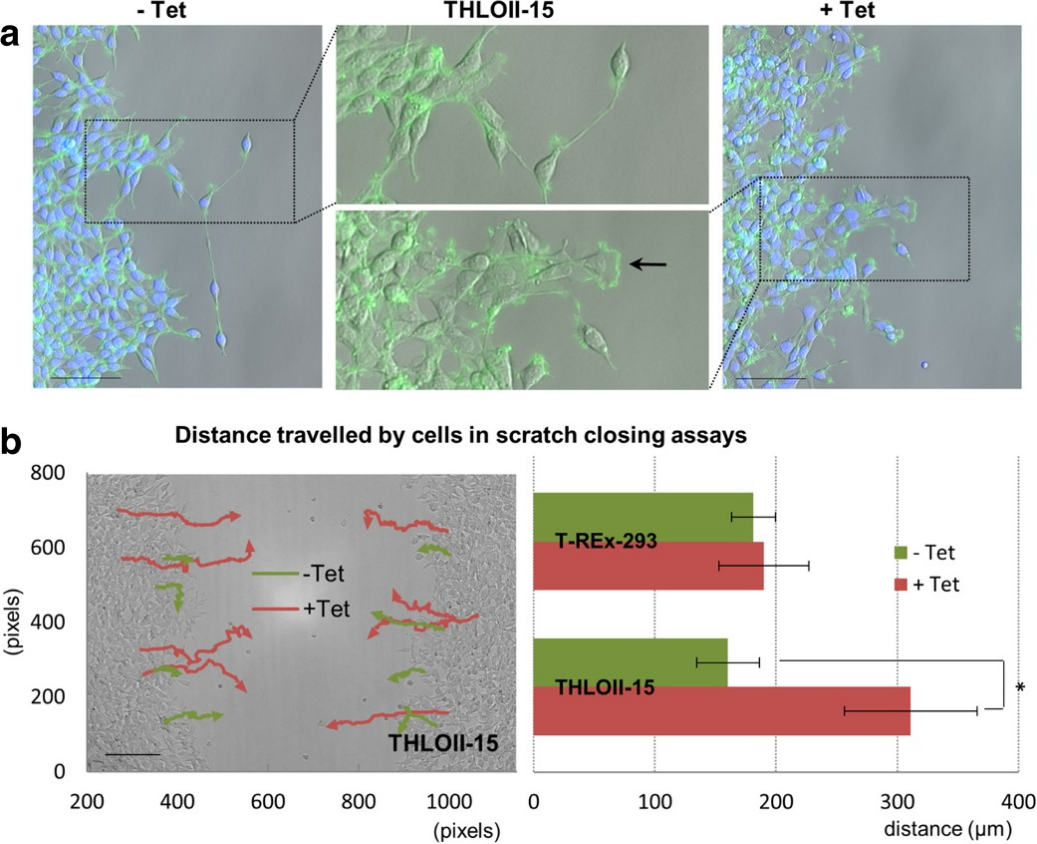
**Enhanced locomotion induced by tetracycline in T-REx-293 cells engineered with** ***Crk-II.*** **(a)** DAPI staining (blue) and phalloidin-FITC (green) during scratch closing assays showing enhanced lamellipodia formation in THLOII-15 cells (a representative clone of T-REx-293 cells carrying the locomotion module) when induced with tetracycline: the induced cells show more frequent formation of lamellipodia, shown in green. Scale bars: 100 μm. **(b)** Distance travelled by cells in scratch closing assays with or without induction of the locomotion module. Images were acquired every 10 min and coordinates of 4 groups of cells on each side of wounds were recorded (6 to 18 h post-induction). Tetracycline increased the migration of THLOII-15 cells (trail plot and graph, *p <0.001) but had no effect on wild-type cells (graph). Statistical analysis was performed using a two-sample Student’s *t*-Test with a two-tailed distribution. Standard deviation bars: n = 8.

A common way of studying cell locomotion is a scratch assay, in which a strip of cells is removed from a monolayer and the behaviour of remaining cells can be observed as they re-colonize the bare zone. Wild-type T-REx-293 cells (Additional file 2: Movie 2, Additional file 3: Movie 3) and T-REx-293 cells containing the locomotion module but with no induction (Additional file 4: Movie 4), showed only modest locomotory activity; some cells occasionally presenting a lamellipodium detectable by phalloidin staining for actin filaments (Figure 6a). When induced, cells expressing CRK-II exhibited enhanced formation of lamellipodia (Figure 6a, Additional file 5: Movie 5), repopulated the scratched area significantly faster and showed almost doubled motility (Figure 6b): the motility of CRK-II-expressing cells increased by a factor of 1.94, with p < 0.001 by Student’s *t*-Test. Wild-type T-REx-293 cells did not show enhanced motility in the presence of tetracycline.

## Discussion

In this study, we describe a synthetic library of five separate morphogenetic effector modules that induce five morphological behaviours in T-REx-293 cells. We have made these biological parts available to others by depositing them in a plasmid repository (Addgene) as pENTR vectors. We have chosen the T-REx-293 cell line as our engineering chassis for its ease of transfection and its built-in tetracycline transcriptional control. It would be interesting to find out how easily our tools can be engineered in cell types that are more problematic to transfect or where sustained transgene expression is difficult to achieve. It would also be useful to know how reliably our effectors perform in other cell lines. The constructs assembled here do not contain cell-type-specific features and should therefore be functional in other mammalian cells. Importantly, the cytomegalovirus (CMV) promoter (used in the T-REx system) has been reported to show poor activity in some cell lines [43] and promoters such as elongation factor 1α (EF1α) could improve transgene expression if needed [44]. This might be particularly relevant when engineering stem cell chassis where CMV has been shown to have poor transcriptional activity [45]. The resultant stem cells (i.e. stably transfected with functional and inducible morphogenetic effectors) could offer new options for synthetic approaches to tissue-engineering.

Also, we have used a simple controlling switch in this study to demonstrate the basic functions of the modules in a synthetic biology context. Better performance may come from more sophisticated switches, for example of the type described by Deans *et al.* [46], that combine RNAi and repressor proteins to improve the leakiness associated with the tetracycline switch. Using switches with translational or post-translational control might also change the kinetics of the system and confer a faster response to environmental cues. In addition, using different promoters may allow for better tunability over the desired morphologic changes in engineered mammalian cells. Also, IRES-dependent expression of fluorescent markers was extremely variable, when expression from 2A constructs was robust and reliable. Exploring the use of other IRESes than the encephalomyocarditis virus (EMCV) IRES used in this study might help to get reliable fluorescent marker expression from bicistronic vectors [47].

We are currently combining sequences of morphogenetic effectors in a series of proof-of-concept trials for synthetic morphology, where cells are genetically programmed to organize themselves into designed 2D or 3D structures in response to artificial external stimuli. To this end, several morphogenetic effectors can be combined in the same chassis, with each effector placed under the control of a different switch to ensure they can be induced at different times or rates.

## Conclusions

There are two broad fields in which the modules described here (and others like them) may be useful, particularly if placed under the control of existing sensing and logic modules [48, 49]. The first is the testing of fundamental theories of developmental biology. There is a strong thread of philosophical thought, traceable to Giambattista Vico (1668-1744), that we can be sure we understand something only if we can (re)create it: synthetic biologists often quote Richard Feynmann’s re-statement of this principle as ‘what I cannot create, I do not understand’. Analytical biology has produced models of how morphogenesis works in simple tissues: using synthetic morphological modules to try to teach naïve cells to undergo similar morphogenesis is a powerful mean of testing the quality of these modules [7]. The second field in which these modules may be useful is that of tissue engineering, especially adapting human cells to live in culture, whether for life-support machines or as test beds for pharmaceutical efficacy or toxicity screening [50–52]. Combinations of various cell lines with different morphologic effectors (the ones described here and others yet unpublished) could be assembled. Importantly, the resulting cell arrangements would not be restricted to natural ones and un-natural, designed structures could be obtained and may, in the future, be used in surgery and regenerative medicine, making synthetic morphology a powerful tool for tissue engineering.

## Methods

### Constructs

Mouse *Cdh1* was amplified from plasmid pBATEM2 [BCCM/LMBP #3585] [53]; mouse *Casp2* was amplified from plasmid pCMF2E-mCASP-2 [BCCM/LMBP #4588] [54], reptilian orthoreovirus *p14* [GenBank: AY238887] was custom-synthesised (Integrated DNA Technologies), *Crk-II* was amplified from adult mouse kidney cDNA and human *p27*^*kip1*^ was amplified from plasmid pcDNA3.1_p27 kindly provided by Bruno Amati [55]. Effector genes were PCR amplified with primers containing attachment (att) sites for Gateway® recombination according to manufacturer’s instructions and recombined by BP reaction in vector pDONR-221 kindly donated by Agnès Roure [56]. The resulting pENTR vectors were deposited to the Addgene plasmid repository. Effector genes were inserted into pT-REx-DEST30 (Invitrogen), pTREx-DEST-IRES-TGFP or pTREx-DEST-IRES-mCherry vectors by LR reaction according to manufacturer’s instructions. pTREx-DEST-IRES-TGFP and pTREx-DEST-IRES-mCherry vectors were generated by inserting NheI/MfeI IRES-TurboGFP or IRES-mCherry cassettes in pT-REx-DEST30. The TurboGFP and mCherry genes were cloned from pTurboGFP-N (Evrogen) and pCherryPicker2 (Clontech), respectively. Plasmid pTREx-mCherry was created by inserting the mCherry gene into pT-REx-DEST30.

### Cell culture, transfections and clonal selection

T-REx-293 cells (Invitrogen) were maintained in T-REx-293 culture medium, which consisted of DMEM (Gibco 41966) supplemented with 10% fetal bovine serum (FBS, Biosera) and 5 mg/mL blasticidin (Gibco), at 37°C and 5% CO_2_. MDCK cells were maintained in MEM medium (Sigma) supplemented with 5% FBS and 1% Penicillin/Streptomycin/L-Glutamine (Sigma). T-REx-293 cells were transfected in 24-well plates using 1 μg effector plasmids and 2 μL lipofectamine 2000 (Invitrogen), in 100 μL Opti-MEM (Gibco) for each well. Cells were then selected using 800 mg/mL G418 (Sigma) for 2 weeks. Stably transfected cells were maintained and tested in T-REx-293 culture medium with 200 mg/mL G418. Individual clones were isolated by plating stably transfected cells in 10-cm cell culture dishes (Corning) at a density of 300-500 cells/dish and cultured for 7-10 days (until individual colonies reached over 100 cells in size). Colonies were picked using cloning cylinders (Bellco) and expanded. For adhesion clones, 1 μg/mL tetracycline (Sigma) was added prior to selection and only GFP-positive colonies were picked. After clonal selection and expansion, all clones were tested for their response to tetracycline induction (1 μg/mL tetracycline for 24 to 48 h). For the adhesion and locomotion modules, clones were selected according to their GFP/mCherry fluorescence intensity and homogeneity (clones THAD1-34 and THLOII-15 were chosen for further assays). For the elective cell death and fusion modules, clones were selected according to the level of cell death or fusion observed (clones THAP2-2, and THFU-4, -9 and -10). For the proliferation control module, clones were selected according to their GFP fluorescence intensity and growth rate (clones THGA-6 and -17). When testing the elective cell death module, the caspase inhibitor Q-VD-OPh (Sigma) was added to the medium (50 μM). The fusion module was tested in regular T-REx-293 culture medium or in calcium-free medium (Gibco 21068) supplemented with 2 mM L-glutamine (Gibco 25030), 10% FBS and 5 mg/mL blasticidin.

### Cell imaging

Live cell images were acquired using a Zeiss AxioObserver D1 inverted fluorescence microscope with AxioCam MRm and a 10× objective. Filter excitation (EX) and emission (EM) bandpass specifications were as follows (in nm): GFP (EX: 470/40, EM: 525/50); mCherry (EX: 545/25, EM: 605/70). Immunostaining images were acquired using the previous Zeiss AxioObserver or the Zeiss AxioImager A1 upright fluorescence microscope with AxioCam MRm and 10x or 20x objectives. Filter excitation (EX) and emission (EM) bandpass specifications were as follows (in nm): DAPI (EX: 365, EM: 445/50); FITC (EX: 450/90, EM: 515/65). For phalloidin immunostaining, cells were seeded on sterile coverslips or in culture plates directly and treated with 1 μg/mL tetracycline for 24 h before fixation with 4% paraformaldehyde. After fixation, cells were permeabilized with 0.1% Triton X-100 and incubated with 0.1 μg/mL FITC-conjugated phalloidin (Sigma) for 1 h at room temperature. Next, cells were stained with 300 nM 4′,6-Diamidino-2-Phenylindole (DAPI) and mounted on glass slides with Vectashield Hardset mounting media (Vector) for fluorescence microscope imaging, when not imaged directly in wells.

### Scratch closing assay

Cells were seeded in 2 mL medium in duplicated 6-well plates. At 90% confluence, cells were scratched and 1 μg/mL tetracycline was added. Time-lapse recordings were performed on LumaScopes 500 (Etaluma, Inc. Carlsbad, CA) with 20x objectives at 37°C and 5% CO_2_. Induced and uninduced wells were imaged in parallel every 10 min for 24 h. Images were captured in the native LumaView software, and ImageJ was used to follow the distance travelled by cells. Four small groups of cells on each side of the scratch were marked image per image, and their coordinates used to calculate the distance travelled between 6 to 18 h after induction. Statistical analysis was performed using a two-sample Student’s *t*-Test with a two-tailed distribution. Multiple scratch closing assays were conducted showing similar results; representative single assays (n = 8) were chosen to illustrate these results in Figure 6b.

### Cell counts

To test the proliferation control module, cells were seeded in 6-well plates and induced with 0.05 μg/mL tetracycline for 48 h before replacing the growth medium with tetracycline-free medium. Adherent cell numbers were determined at different times after induction and after release from induction: at each time point, cells from triplicate wells were harvested by trypsinization after removal of growth medium (containing negligible numbers of floating dead cells), and cells were counted on haemocytometers. To test the elective cell death module, cells were seeded in 24-well plates and induced with 1 μg/mL tetracycline. Adherent cell numbers were determined at different times before and after induction as described above. To quantify the effect of the Q-VD-OPh caspase inhibitor on induced cell death, images of cell culture 48 h after induction were analysed on ImageJ: cell numbers were estimated by measuring the area covered by remaining adherent cells in duplicate image fields.

### RT-PCR

mRNA was extracted from wild-type T-REx-293 cells and from clone THAD1-34 cells induced or uninduced with tetracycline for 48 h. Briefly, cells were collected at confluence from a 6-well plate and mRNA was extracted following RNeasy kit’s instructions (QIAGEN) with an additional DNA digestion step with DNase (QIAGEN). RT-PCR was performed on 2 μg of RNA with MLV-RT (Promega). cDNAs from *Cdh1* and *β-actin* as internal control were amplified with specific primers over 20 PCR cycles. Primers for mouse *Cdh1*: Cdh1_129F (TGCCGGAGAGGCACCTGGAG) and Cdh1_391R (GGTGGTGGTGCCGGTGATGG); primers for *β-actin*: β-actin_F (CTGGGACGACATGGAGAARA) and β-actin_R (AAGGAAGGCTGGAARAGWGC)].

## Electronic supplementary material

Additional file 1: Movie 1: Time-lapse imaging of cells from clone THAP2-2 undergoing tetracycline-induced elective cell death (images captured every 30 min for 70 h after addition of Tet). (MP4 10 MB)

Additional file 2: Movie 2: Time-lapse imaging of wild-type T-REx-293 cells in control scratch closing assay without tetracycline (images captured every 10 min for 12 h). (MP4 6 MB)

Additional file 3: Movie 3: Time-lapse imaging of wild-type T-REx-293 cells in scratch closing assay with 1 μg/mL tetracycline (images captured every 10 min for 12 h: 6 to 18 h after addition of Tet). (MP4 6 MB)

Additional file 4: Movie 4: Time-lapse imaging of cells from clone THLOII-15 in control scratch closing assay without tetracycline (images captured every 10 min for 12 h). (MP4 6 MB)

Additional file 5: Movie 5: Time-lapse imaging of cells from clone THLOII-15 in scratch closing assay with 1 μg/mL tetracycline (images captured every 10 min for 12 h: 6 to 18 h after addition of Tet). Notes: All movies have the same spatial scale (1 mm across) and run at the same speed for ease of comparison, except for Movie 1 which runs three times faster. (MP4 5 MB)

## Abbreviations

2D: Two-dimensional
3D: Three-dimensional
GFP: Green fluorescent protein
HEK: Human embryonic kidney
IRES: Internal ribosome entry site
MDCK: Madin-Darby canine kidney
Tet: Tetracycline.
